# Covalent Organic Framework-Based Nanomembrane with Co-Immobilized Dual Enzymes for Micropollutant Removal

**DOI:** 10.3390/nano15181431

**Published:** 2025-09-18

**Authors:** Junda Zhao, Guanhua Liu, Xiaobing Zheng, Liya Zhou, Li Ma, Ying He, Xiaoyang Yue, Yanjun Jiang

**Affiliations:** 1 Arizona College of Technology, Hebei University of Technology, Tianjin 300401, China; jundazhao@arizona.edu; 2 School of Chemical Engineering and Technology, Hebei University of Technology, Tianjin 300401, China; ghliu@hebut.edu.cn (G.L.); zhengxiaobing@hebut.edu.cn (X.Z.); liyazhou@hebut.edu.cn (L.Z.); mali0502@hebut.edu.cn (L.M.); yanjunjiang@hebut.edu.cn (Y.J.)

**Keywords:** biocatalytic membrane, immobilized enzyme, micropollutant removal

## Abstract

Biocatalytic nanomembranes have emerged as promising platforms for micropollutant remediation, yet their practical application is hindered by limitations in removal efficiency and operational stability. This study presents an innovative approach for fabricating highly stable and efficient biocatalytic nanomembranes through the co-immobilization of horseradish peroxidase (HRP) and glucose oxidase (GOx) within a covalent organic framework (COF) nanocrystal. Capitalizing on the dynamic covalent chemistry of COFs during their self-healing and self-crystallization processes, we achieved simultaneous enzyme immobilization and framework formation. This unique confinement strategy preserved enzymatic activity while significantly enhancing stability. HRP/GOx@COF biocatalytic membrane was prepared through the loading of immobilized enzymes (HRP/GOx@COF) onto a macroporous polymeric substrate membrane pre-coated with a polydopamine (PDA) adhesive layer. At HRP and GOx dosages of 4 mg and 4.5 mg, respectively, and a glucose concentration of 5 mM, the removal rate of bisphenol A (BPA) reached 99% through the combined functions of catalysis, adsorption, and rejection. The BPA removal rate of the biocatalytic membrane remained high under both acidic and alkaline conditions. Additionally, the removal rate of dyes with different properties exceeded 88%. The removal efficiencies of doxycycline hydrochloride, 2,4-dichlorophenol, and 8-hydroxyquinoline surpassed 95%. In this study, the enzyme was confined in the ordered and stable COF, which endowed the biocatalytic membrane with good stability and reusability over multiple batch cycles.

## 1. Introduction

Various micropollutants (MPs), including antibiotics and endocrine disruptors, are commonly found in industrial and medical wastewater [1]. These micropollutants are harmful to both human health and the ecological environment, affecting the reproductive, nervous, and immune systems of humans and animals [2,3]. MPs typically exist in water at trace concentrations ranging from ng/L to μg/L scales, which complicates detection and analysis, and poses challenges for water treatment processes [4]. Bisphenol A (BPA), a prevalent microplastic, is toxic to humans, disrupting sex hormones, insulin, and thyroxine while causing liver damage, immune dysfunction, and cancer risks [5,6,7,8,9].

MPs pose formidable control challenges due to their persistent toxicity, environmental stability, and diverse contamination sources. Therefore, MPs require urgent control to reduce their negative impact on both the environment and humans. Current removal strategies include: (1) physical (membrane filtration, adsorption), (2) chemical (advanced oxidation, photocatalysis), and (3) biological (enzymatic degradation) approaches [10,11,12]. However, these methods still have limitations. For instance, physical techniques like adsorption, membrane separation, and flocculation often generate secondary waste or struggle with small MPs; chemical methods (e.g., Fenton oxidation, photocatalysis) are energy-intensive and may produce toxic byproducts; biodegradation is slow and selective. Therefore, to develop more efficient technologies is very urgent.

Oxidoreductases can oxidize phenolic compounds, including various drugs and antibiotics, to form free radicals or products such as dimers and trimers, which are generally less toxic than the original phenolic compounds [13,14,15,16,17]. Horseradish peroxidase (HRP, EC1.11.1.7) is a glycoprotein complex oxidase with an iron porphyrin cofactor, produced by microorganisms or plants, mainly derived from horseradish roots. Hydrogen peroxide (or other peroxides) is required as a final electron acceptor to oxidize substrates [18]. HRP has been widely used in the treatment of phenolic MPs. It can convert BPA into an insoluble polymer, which can be removed from water through simple precipitation and filtration [19,20].

The immobilization of the enzyme on a membrane to form a biocatalytic membrane enhances the enzyme’s environmental tolerance, enables reusability, and allows for the removal of degradation products [21,22]. However, conventional immobilization on flat membranes suffers from low enzyme loading due to limited surface area. Instead, utilizing porous membrane structures with high surface area significantly improves co-immobilization capacity and catalytic performance [23]. In recent years, biocatalytic membranes, which combine physical separation and biological catalysis, have been considered as a promising method for the removal of MPs [24,25,26].

Currently, widely used amorphous polymer membranes suffer from disordered structures and nonadjustable pores. On the other hand, crystalline covalent organic frameworks (COFs) offer ordered networks with precisely tunable pores, high porosity, and large surface areas [27,28]. Therefore, COF membranes significantly advance separation technologies by combining precise porosity with multifunctionality. Ma et al. developed a biocatalytic membrane through in situ ZIF-8 biomineralization for horseradish peroxidase (HRP) immobilization, achieving 98% removal of bisphenol A (BPA) via enzymatic oxidation [29]. Zhang et al. designed a glucose oxidase (GOx)/HRP co-immobilized polyamide membrane capable of tunable cascade catalysis regulated by glucose concentration and transmembrane pressure. This system attained efficient BPA removal (>90%) under optimal conditions [30]. Despite these promising results, the broader application of enzyme-immobilized porous polymer materials still faces significant challenges, such as pore tunability and difficulties in achieving uniform enzyme distribution and long-term retention of activity.

The crystallization process of imine-linked COFs proceeds through an initial formation of low-crystalline polymeric intermediates, which subsequently undergo gradual structural ordering into well-defined crystalline frameworks [31,32]. This transformation process offers a potential strategy for enzyme encapsulation and stabilization. [33,34,35]. The enzymes are first entrapped within the mesoporous amorphous COF (aCOF) during initial growth, gaining protection from harsh environments. Subsequent self-repair and self-crystallization process of COF then permanently encapsulates the enzymes within the ordered framework.

Here in this study, HRP and GOx were in situ encapsulated in COF using this strategy, which not only protects enzymes via the initial formation of low-crystalline enzyme-polymer intermediates, but also enables the encapsulation of enzymes with larger size, well preserving the activity and enhancing the stability of enzyme. The prepared HRP/GOx@COF was then employed to fabricate a biocatalytic membrane via polydopamine-assisted self-assembly method, achieving efficient MPs removal through the synergistic effect of catalysis, rejection, and adsorption. Although enzyme immobilization has been successfully achieved using porous crystalline frameworks such as MOFs and COFs as carriers, the current approaches mostly rely on post-synthetic encapsulation methods. In contrast, our self-crystallization approach involves the co-assembly of COF precursors and enzymes during framework formation, enabling a more homogeneous distribution and higher loading efficiency. This approach substantially reduces mass transfer constraints and improves enzymatic stability by leveraging a customized pore, demonstrating clear benefits compared to traditional encapsulation methods.

## 2. Materials and Methods

### 2.1. Materials and Chemicals

1,3,5-Tri(4-aminophenyl)benzene (TAPB, 97.0%) and 2,5-dimethoxy-p-phenyldiformaldehyde (97.0%) were provided by Jilin Scientific Research Extension Technology Co., Ltd (Changchun, China). Acetonitrile (AR) was supplied by Tianjin Kermel Chemical Reagent Co., Ltd (Tianjin, China). Sodium hydroxide (NaOH, 96.0%), dopamine hydrochloride (98.0%), and HRP (EC 1.11.1.7) were provided by Shanghai Aladdin Biochemical Technology Co., Ltd (Shanghai, China). GOx (EC 1.1.3.4) was supplied by Shanghai Yuanye Biotechnology Co., Ltd. (Shanghai, China) Polyacrylonitrile (PAN) ultrafiltration membrane (MWCO = 50 kDa) was manufactured by Guochu Technology (Xiamen) Co., Ltd (Xiamen, China). Hydrochloric acid (HCl, 37 wt.%) was supplied by Tianjin Jinhai Huaxing Co., Ltd (Tianjin, China).Doxycycline hydrochloride (DC), tetracycline hydrochloride (TC), 2,4-dichlorophenol (2,4-DCP), 8-hydroxyquinoline (8-HQ), p-nitrophenol (4-NP), and hydroquinone (HQ) were provided by Shanghai Aladdin Biochemical Technology Co., Ltd. (Shanghai, China).

### 2.2. Synthesis of Amorphous COF (aCOF)

The synthesis of aCOF was performed using a previously reported method with some adjustments [36]. 1,3,5-Tris(4-aminophenyl)benzene (TAPB, 35.3 mg, 0.1 mmol), 2,5-dimethoxyterephthalaldehyde (29.3 mg, 0.15 mmol), and 12 mL of acetonitrile (ACN) were placed into a centrifuge tube and sonicated for 1 min at room temperature to completely dissolve the monomers. The mixed solution was then treated with 2.4 mL of acetic acid (6 M), placed at 25 °C for 20 min, and centrifuged to obtain a yellow precipitate. The precipitate was then washed three times with acetonitrile (ACN) and ultrapure water, respectively, to obtain aCOF. The synthesis was highly reproducible across multiple batches (*n* = 3), with the typical mass yield of the COF product being 85 ± 5%, based on the total mass of the initial organic building blocks.

### 2.3. Synthesis of HRP/GOx@aCOF

First, 200 μL of PBS buffer (pH 6.5, 10 mM) containing HRP and GOx was added to 3 mL of PBS buffer (10 mM) containing pre-dispersed aCOF (10 mg). The resulting mixture was shaken at 500 rpm at room temperature overnight. The product was then centrifuged and washed three times with ultrapure water to remove any loosely adsorbed HRP/GOx, resulting in HRP/GOx@aCOF.

### 2.4. Synthesis of HRP/GOx@COF

First, 3 mL of ACN was added to the centrifuge tube containing the obtained HRP/GOx@aCOF. Then, 0.6 mL of acetic acid (6 M) was added to the mixture. The resulting mixture was placed at 25 °C for 6 h, and the final product was washed twice with ACN and ultrapure water, respectively. The preparation method for COF was the same as for HRP/GOx@aCOF, except that enzymes were not added.

### 2.5. Synthesis of Biocatalytic Membrane

The PAN ultrafiltration membrane was hydrolyzed in a 1 M NaOH solution at 50 °C for 1 h, and the residual NaOH was removed with deionized water to obtain the hydrolyzed polyacrylonitrile (HPAN) membrane. The HPAN membrane was then placed in the ultrafiltration cup with the separation layer facing up. Subsequently, 20 mg of dopamine hydrochloride was dissolved in 10 mL of Tris-HCl buffer (25 mM, pH = 8.8) and mixed ultrasonically. The reaction solution was introduced into the device and incubated at 37 °C with shaking for 4 h. Subsequently, the mixture was decanted, and the membrane was thoroughly rinsed with ultrapure water to eliminate residual dopamine monomers, yielding the polydopamine-coated HPAN (PDA@HPAN) membrane. Then, pour the HRP/GOx@COF solution into the device and filter at 0.1 MPa until no droplets remain. Finally, the obtained HRP/GOx@COF membrane was removed and gently washed three times with deionized water.

### 2.6. Membrane Performance Evaluation

#### 2.6.1. Pure Water Permeability

The membrane to be tested was first mounted in the ultrafiltration cup with the selective layer oriented upward. It was then compacted at 2 bar for 20 min to achieve steady-state hydraulic conditions. After stabilizing the operation pressure at 1 bar, permeate samples were collected at predetermined time intervals. Each membrane was subjected to a minimum of three independent filtration trials to ensure reproducibility. The pure water permeance (*PWP*, L m^−2^ h^−1^ bar^−1^ or LMH/bar) was calculated using the following formula:

(1) $$PWP=\frac{V}{A\times t\times P}$$

where *V* (L) represents the total volume of pure water penetrating in a given time *t* (h), *A* (m^2^) is the effective membrane area (2.72 × 10^−3^ m^2^), and *P* (bar) is the operating pressure.

#### 2.6.2. Dye Rejection

A standard curve of concentration versus absorbance was plotted by measuring the absorbance of dye solutions with varying concentrations at their maximum absorption wavelengths: 668 nm for MEB, 614 nm for MG, 530 nm for CBT, 504 nm for CR, and 600 nm for MB. A 10 mg/L dye solution was used to test dye rejection using the ultrafiltration cup at 1 bar. The absorbance of the permeate solution was measured with an ultraviolet-visible (UV–vis) spectrophotometer, and the concentration was calculated using the standard curve mentioned above. The dye rejection (*R*, %) is calculated as follows:

(2) $$R=\left(1-\frac{C_{p}}{C_{f}}\right)\times 100\%,$$

where *C_p_* and *C_f_* are the concentrations (mg/L) of permeate and feed solutions, respectively.

#### 2.6.3. MP Removal Rate

A standard curve of BPA concentration versus corresponding peak areas was plotted through the analysis of BPA solutions with varying concentrations via high-performance liquid chromatography (HPLC). The chromatographic column used was a C18 column (Eclipse Plus C18, 250 mm × 4.6 mm, 5 μm, Agilent). The analytical conditions were set as follows: mobile phase of methanol: water (8:2), flow rate of 1 mL/min, sample injection volume of 20 μL, and column oven temperature of 30 °C. The detection wavelength for the samples was 278 nm, and the sample retention time was 4.78 min. A 25 mL BPA solution (10 mg/L) was added to the ultrafiltration cup, and the permeate was collected within 4 h at room temperature (25 °C). The enzymatic reaction was quenched by adding 0.1 M HCl. The residual BPA concentration was analyzed by HPLC, and the removal efficiency (*R*, %) was calculated according to Equation (2).

The removal efficiencies of other micropollutants, including DC (50 mg/L), HQ (20 mg/L), 2,4-DCP (20 mg/L), TC (50 mg/L), 4-NP (20 mg/L), and 8-HQ (50 mg/L) were comparable to that of BPA, with the following exceptions: For DC, the mobile phase was methanol: water (9:1) with a detection wavelength of 280 nm. For HQ, the mobile phase was acetonitrile: water (1:1). For 2,4-DCP, the mobile phase was methanol: water (7:3) with a detection wavelength of 285 nm. For TC, the mobile phase was acetonitrile: water (1:3) with a detection wavelength of 280 nm. For 4-NP, the mobile phase was methanol: water (7:3) with a detection wavelength of 220 nm. For acetaminophen (APAP), the mobile phase was methanol: water (35:65) with a detection wavelength of 243 nm. The concentration of 8-HQ was measured using a UV–vis spectrophotometer at 625 nm.

### 2.7. Enzyme Activity of Biocatalytic Membrane

#### 2.7.1. The Protein Immobilization Yield

The protein immobilization yield was determined by quantifying unbound enzyme in the supernatant post-synthesis. Briefly, the supernatant was collected after centrifugation (12,000 rpm, 10 min) and analyzed via the Bradford assay (λ = 595 nm). The immobilization yield was calculated as:

$$Immobilization\;yield\;\left(\%\right)=\frac{C\left(initial\right)-C\left(supernatant\right)}{C\left(initial\right)}\times 100\%,$$

where the *C*(*initial*) and *C*(*supernatant*) refer to the enzyme concentrations before and after immobilization.

#### 2.7.2. The Membrane Surface Enzyme Activity

First, 2 mL of guaiacol (15 mM), and 1 mL of PBS buffer (pH = 6) were mixed under agitation. The absorbance (*A*_0_) at 470 nm was measured. HRP was added to the solution and allowed to react for 2 min, followed by measurement of the absorbance (*A*_1_). The membrane surface enzyme activity (*U*_HRP_) was calculated using Equation (3).

(3) $$U_{HRP}=\frac{A_{1}-A_{0}}{T\times \tau \times M}\times V,$$

where *T* is the reaction time (min), *τ* is the molar absorption coefficient (26.6 mL μmol^−1^ cm^−1^), and *M* is the actual amount of enzyme (mg).

### 2.8. Stability and Reusability Test

The storage stability of the biocatalytic membrane was evaluated by measuring the BPA removal rate after specific storage periods in ultrapure water. The testing method was the same as for the performance evaluation. The membrane’s reusability was assessed by measuring its BPA removal rate after each reuse cycle. After each cycle, the membrane surface was thoroughly cleaned with ultrapure water three times.

### 2.9. Sample Preparation for Characterization

Fourier Transform Infrared Spectroscopy (FTIR): FT-IR spectra were recorded on a Bruker GMBH VERTEX 70 spectrometer equipped with a DTGS detector. Samples were prepared by mixing the dry powder with anhydrous KBr (1:100 weight ratio) and pressing into a pellet. Spectra were acquired in transmission mode over the range of 4000–400 cm^−1^ with a resolution of 4 cm^−1^ and 64 scans per sample. Background scans were collected prior to each measurement.

X-ray Photoelectron Spectroscopy (XPS): XPS analysis was performed on a Thermo Fisher Technologies-ESCALAB 250Xi spectrometer using a monochromatic Al Kα X-ray source (1486.6 eV). Powder samples were mounted on double-sided carbon tape. Survey and high-resolution spectra were collected with a pass energy of 150 eV and 50 eV, respectively, and a step size of 1.0 eV and 0.1 eV, respectively. Charge compensation was applied using a dual-beam flood source. All binding energies were referenced to the adventitious C 1s peak at 284.8 eV.

Atomic Force Microscopy (AFM): AFM measurements were performed on a Bruker Dimension Icon microscope in PeakForce Tapping mode using ScanAsyst-Air probes (Bruker). Samples were prepared by dispersing the powder in ethanol, sonicating for 15 min, and drop-casting onto a freshly cleaved mica substrate. Images were processed and analyzed using Gwyddion software (v. 2.62); the root mean square (RMS) roughness was calculated from at least three different 5 × 5 μm scans.

X-ray Diffraction (XRD): Powder XRD patterns were collected on a Bruker D8 Advance diffractometer using Cu Kα radiation (*λ* = 1.5406 Å) operated at 40 kV and 40 mA. Data were recorded in the 2θ range of 2–30° with a step size of 0.02° and a counting time of 1 s per step. Samples were prepared as a thin layer on a zero-background silicon holder.

## 3. Results and Discussion

### 3.1. Characterization of COF Nanoparticles and Immobilized Enzyme

**Figure 1:**
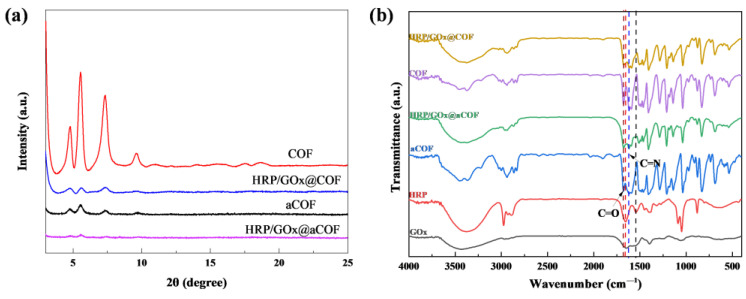
(**a**) XRD patterns of aCOF, COF, HRP/GOx@aCOF and HRP/GOx@COF, (**b**) FT-IR spectra of COF, HRP, GOx and immobilized enzymes.

As shown in Figure 1a, the crystal structures of aCOF and COF were analyzed via XRD. The diffraction peaks of aCOF and COF at 4.65°, 5.41°, 7.26°, and 9.48° were consistent with those reported in the literature [36,37], indicating that COF had good crystallinity. The weak peaks in the diffraction patterns of HRP/GOx@COF also revealed the influence of enzyme on the self-healing and self-crystallization processes of COF.

The chemical compositions of HRP, GOx, aCOF, HRP/GOx@aCOF, COF, and HRP/GOx@COF were analyzed via FTIR spectroscopy. As shown in Figure 1b, the stretching vibration peak of the C=N bond at 1617 cm^−1^ indicated the formation of the imine bond, confirming the successful synthesis of COF [33,35]. The stretching vibration peak of the C=O bond at 1680 cm^−1^ corresponded to the unreacted aldehyde residue [38]. The HRP/GOx@aCOF and HRP/GOx@COF samples exhibited characteristic peaks of HRP and GOx at 1076 cm^−1^, 1542 cm^−1^ and 1652 cm^−1^, respectively, confirming the successful immobilization of the enzymes.

**Figure 2:**
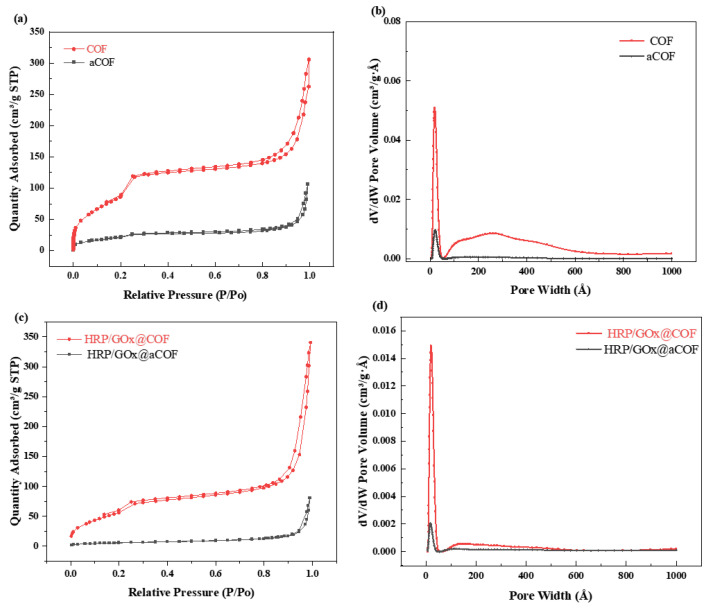
N_2_ adsorption–desorption isotherms and pore size distribution of (**a**,**b**) aCOF, COF and (**c**,**d**) HRP/GOx@aCOF, HRP/GOx@COF.

The pore properties of the carrier directly affected the loading capacity and activity of the enzymes. N_2_ adsorption and desorption experiments were performed on aCOF, COF, HRP/GOx@aCOF, and HRP/GOx@COF to determine their specific surface area, pore volume, and pore size. As shown in Figure 2, both aCOF and COF exhibited type IV isotherms, with N_2_ adsorption increasing sharply at lower pressures, indicating the presence of micropores [39]. The data in the range of 0.1 < P/P_0_ < 0.3 corresponded to the mesoporous structure of COF. The N_2_ adsorption continued to increase as pressure increased (0.4 < P/P_0_ < 1.0), indicating its permanent mesoporous nature [40]. After dual enzyme loading, the specific surface area of both aCOF and COF decreased, as the enzymes occupied a portion of the pore space. The pore size distribution diagram and Table S1 show that the average pore size of aCOF was approximately 10.44 nm, while the dimensions of HRP were 4.0 nm × 4.4 nm × 6.8 nm [41], and those of GOx were 7.0 nm × 5.5 nm × 8.0 nm [42]. The pore size of aCOF was larger than that of the dual enzyme, allowing for enzyme immobilization within the pores through adsorption. Subsequently, the enzyme could be immobilized as aCOF transformed into a highly crystalline and porous COF. The changes in specific surface area and pore size of aCOF, HRP/GOx@aCOF, COF, and HRP/GOx@COF demonstrated the successful immobilization of the enzymes.

**Figure 3:**
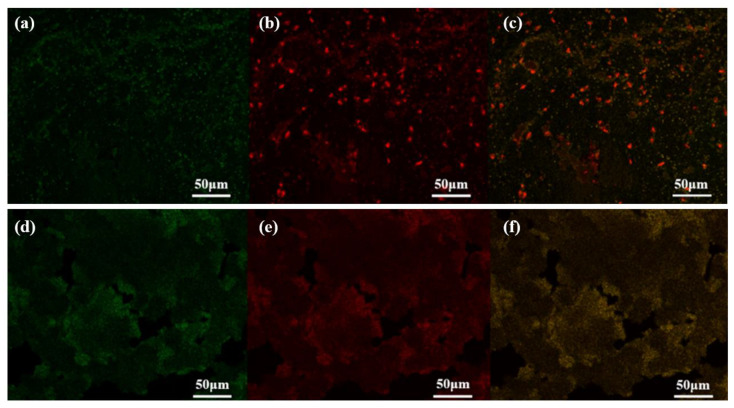
CLSM images of HRP/GOx@aCOF (**a**,**b**) and HRP/GOx@COF (**d**,**e**), HRP and GOx were marked with FITC (green) and RhB (red), respectively, which turned light yellow (**c**,**f**) after overlap.

To observe the distribution of HRP and GOx in the carriers, FITC and RhB were used to stain HRP and GOx, respectively, as both could emit fluorescence upon excitation. Compared with the CLSM images of pristine aCOF and COF in optical (Figure S1), the CLSM images of marked HRP/GOx@COF, in fluorescence (Figure 3) showed that HRP and GOx were evenly distributed within the aCOF and COF, further confirming the successful immobilization of the dual enzymes in the carrier.

**Figure 4:**
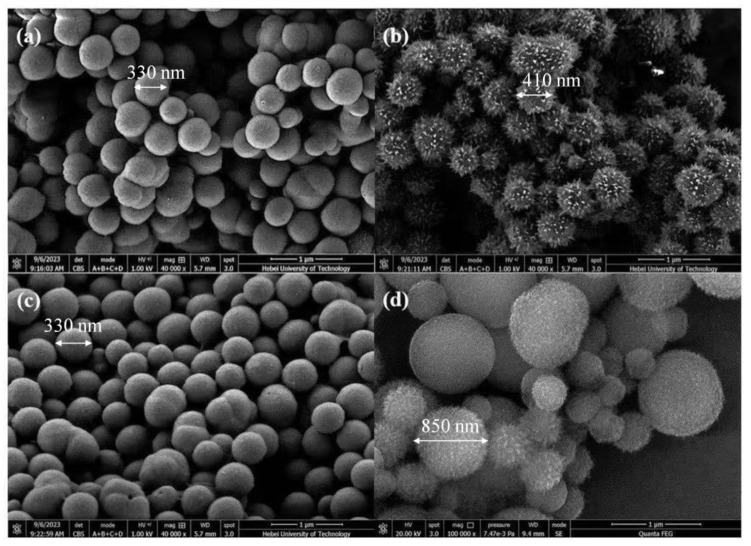
The SEM images of (**a**) aCOF, (**b**) COF, (**c**) HRP/GOx@aCOF, (**d**) HRP/GOx @COF.

**Figure 5:**
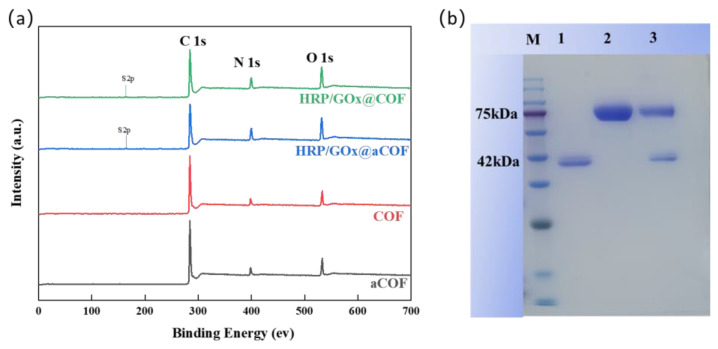
(**a**) XPS survey of aCOF, COF, HRP/GOx@aCOF and HRP/GOx@COF, (**b**) SDS-PAGE image (M, protein maker; lane 1, HRP, lane 2, GOx; lane 3, HRP/GOx@COF).

As shown in Figure 4, aCOF initially exhibited a smooth, spherical shape with a diameter of approximately 330 nm. After undergoing self-healing and self-crystallization, its morphology transformed into a spiny sea urchin-like structure, with an increased diameter of ~410 nm and uniform particle size. Upon enzyme loading, HRP/GOx@aCOF maintained its original spherical structure without substantial morphological changes. However, after the self-crystallization and self-healing process, the surface morphology of HRP/GOx@COF underwent notable alterations: the spiny structures were replaced by short rod-like features, and the particle size changed substantially. This transformation can be attributed to the influence of the encapsulated enzyme proteins, which interfered with the internal self-healing and self-crystallization processes. The enzymes may have interacted with the COF through pore adsorption, potentially modifying crystallization behavior or self-healing capability. By occupying COF pores, the enzymes could restrict or promote molecular movement and arrangement, thereby affecting crystallization. Additionally, the enzymes might have altered the internal chemical environment (e.g., pH, ionic strength), influencing the reactivity of COF functional groups and ultimately leading to the observed morphological changes during self-healing and self-crystallization. Moreover, the presence of sulfur (S) element in XPS spectra further confirms the immobilization of enzymes Figure 5a.

To confirm that HRP and GOx were successfully immobilized in the pores of COF, qualitative protein analysis was performed via SDS-PAGE gel electrophoresis. Figure 5b shows distinct bands at 42 kDa [41] and 75 kDa, corresponding to free HRP (lane 1) and GOx (lane 2), respectively. HRP/GOx@COF (lane 3) displayed bands at 42 kDa and 75 kDa, indicating the successful immobilization of HRP and GOx in the pores of COF. And the amounts of immobilized HRP and GOx were also quantified as 255.1 mg/g_support_ and 441.7 mg/g_support_, respectively, with retained activity of 70% and 65%.

### 3.2. Characterization of Biocatalytic Membranes

**Figure 6:**
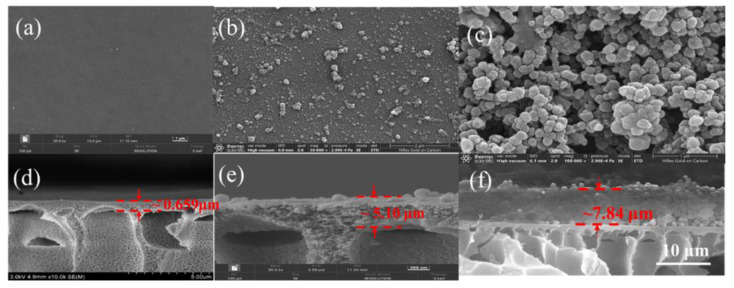
SEM images of surface and cross-section morphology of (**a**,**d**) HPAN, (**b**,**e**) PDA@HPAN, and (**c**,**f**) HRP/GOx@COF membrane.

The surface and cross-sectional morphology of the prepared membranes were observed via SEM. As shown in Figure 6, the surface of the HPAN membrane was smooth. After coating with PDA, some nanoaggregates became visible. The PDA coating exhibited non-specific adhesion owing to its phenolic hydroxyl groups and adhered to material surfaces through non-covalent interactions, such as hydrogen bonding, complexation, and π–π stacking [43,44]. As shown in Figure 6c,f owing to the adhesion of PDA, HRP/GOx@COF nanoparticles were stacked on the surface of the PDA@HPAN membrane. The thickness of the HRP/GOx@COF membrane was 7.84 μm. The AFM results (Figure S2, Table S2) revealed that the HPAN membrane had a smooth surface (Ra = 34.9 nm). After polydopamine coating, roughness slightly increased. Upon immobilizing HRP/GOx@COF nanoparticles, the surface became more uneven (Ra = 85.1 nm), consistent with nanoparticle attachment.

**Figure 7:**
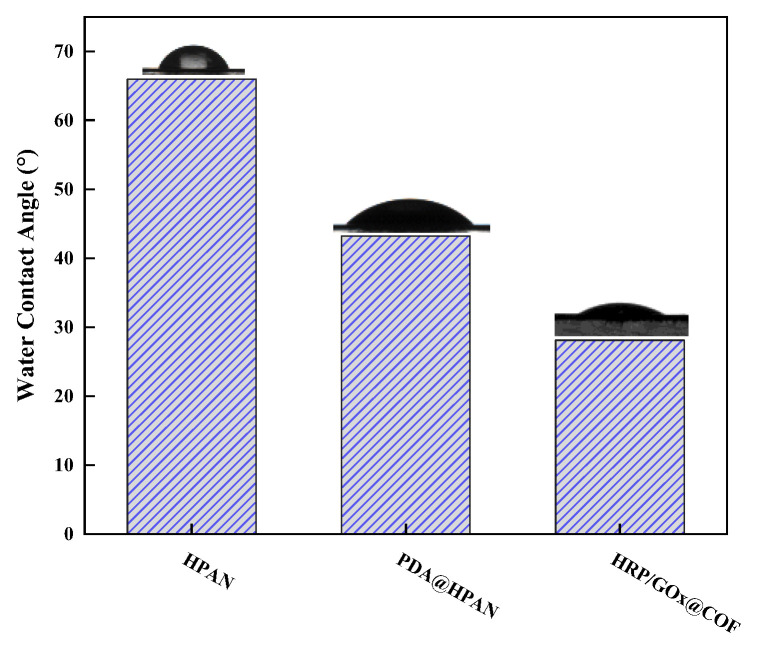
WCAs of HPAN, PDA@HPAN and HRP/GOx@COF membranes.

WCAs were used to evaluate the hydrophilicity of the membranes (Figure 7). The HPAN membrane exhibited hydrophilicity owing to the hydrolysis of the nitrile group to a hydrophilic carboxyl group. After coating with the PDA layer, the WCA decreased, owing to the hydrophilic amine and hydroxyl groups in the PDA. When the HRP/GOx@COF nanoparticles were introduced, the membrane WCA further decreased to 28.1°. This is attributable to the relatively high hydrophilicity of COF and the increased surface roughness of the membrane (Table S2). According to Wenzel’s equation, the WCA of a hydrophilic surface decreases as surface roughness increases. Moreover, although the improved hydrophilicity can lead to a higher water permeance, the modification by PDA and the immobilization of enzymes increased the mass transfer resistance, which caused a lower water permeance of HRP/GOx@COF membrane (66.176 LMH/bar, Figure S3).

### 3.3. Separation Performance of Biocatalytic Membranes

#### 3.3.1. Effect of Dual Enzyme Addition on HRP Enzyme Activity

**Figure 8:**
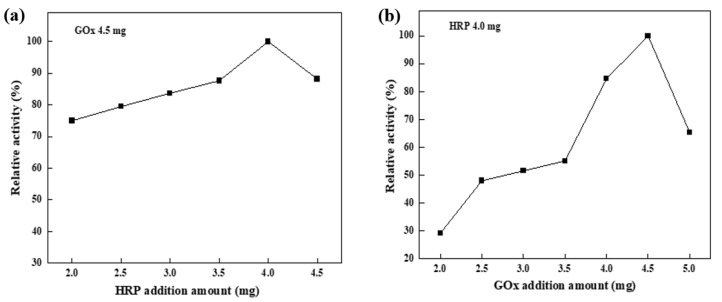
Relative activity of membrane at different addition amount of HRP (**a**) (GOx 4.5 mg) and GOx (**b**) (HRP 4.0 mg). (glucose: 5 mM, temperature: 25 °C, pH: neutral).

The ratio of enzymes in the dual enzyme was crucial factor affecting the removal rate of biocatalytic membranes. As shown in Figure 8a, when the added amount of HRP was low, the substrate oxidation rate was limited because the produced H_2_O_2_ could not be effectively utilized. However, as the amount of HRP increased to 4 mg, the substrate oxidation rate improved, as more HRP was available to catalyze the reaction. However, when an excessive amount of HRP was added, the enzyme’s active sites might overlap, which could lead to a decrease in its apparent activity. As shown in Figure 8b, when the amount of GOx added was low, the glucose oxidation rate was limited, resulting in insufficient H_2_O_2_ production. This directly impacted the HRP-catalyzed substrate oxidation reaction, as HRP required H_2_O_2_ as an electron acceptor. As the amount of GOx added increased to 4.5 mg, both the oxidation rate of glucose and the production of H_2_O_2_ rose accordingly. This provided more electron acceptors for HRP, thereby enhancing the substrate oxidation rate. However, when too much GOx was added, excess H_2_O_2_ was produced, which reduced HRP enzyme activity. The optimal immobilized enzyme amounts were determined to be 4 mg HRP and 4.5 mg Gox (M_HRP_/M_GOx_ = 8:9), which achieved the highest catalytic efficiency in the system. In this case, the amounts of immobilized enzymes per membrane area were quantified as 0.236 mg/cm^2^, with a specific activity of 0.05 U/mg (HRP) and 0.15 U/mg (GOx).

#### 3.3.2. Effect of Glucose Concentration on the Properties of Biocatalytic Membranes

**Figure 9:**
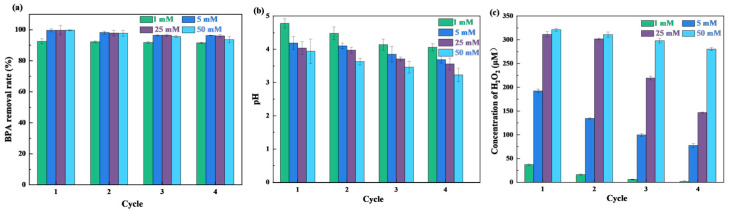
Effect of glucose concentration on (**a**) BPA removal rate by cascade catalysis in HRP/GOx@COF membrane, (**b**) the permeate pH with reuse cycle and (**c**) H_2_O_2_ concentration produced by HRP/GOx@COF membrane. (BPA: 10 mg/L, temperature: 25 °C, pH: neutral).

Figure 9a shows the effect of glucose concentrations on BPA removal. In the first cycle, the biocatalytic membrane removed 92.5% of BPA with 1 mM glucose, while with 5 mM, 25 mM, and 50 mM glucose, all the biocatalytic membranes removed over 99% of BPA. The increase in glucose concentration provided more H_2_O_2_ to the microenvironment within the membrane, enhancing BPA degradation by HRP. Meanwhile, the pH of the system gradually decreased, as shown in Figure 9b. The removal of BPA by the biocatalytic membrane with 1 mM glucose remained largely unchanged with an increasing number of reuse cycles. The removal of BPA with 5 mM, 25 mM, and 50 mM glucose slightly decreased after reuse, likely owing to the inhibitory effect of the excess H_2_O_2_ on HRP activity [31]. Additionally, the concentration of H_2_O_2_ produced by GOx in the system gradually decreased with an increasing number of reuse cycles (Figure 9c), which caused a reduced enzyme activity.

#### 3.3.3. Removal of BPA by Biocatalytic Membranes Under Various Conditions

**Figure 10:**
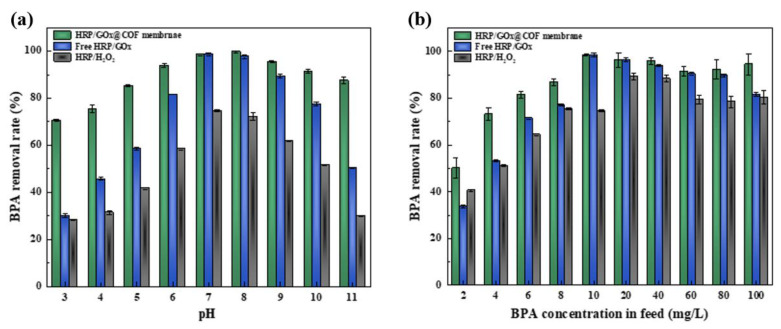
BPA removal rate of HRP/GOx@COF membrane, free HRP/GOx and HRP/H_2_O_2_ at different feed solution pH (**a**) (BPA: 10 mg/L) and different BPA concentration (**b**) (pH: neutral). (glucose: 5 mM, HRP: 4 mg, GOx: 4.5 mg, H_2_O_2_: 3 mM, temperature:25 °C).

The pH of solutions is an important factor in practical wastewater treatment; therefore, the removal of BPA by the biocatalytic membrane at different pH levels was tested. As shown in Figure 10a, the removal rate of BPA by both the biocatalytic membrane and free HRP/GOx remained high, above 80%, at pH values of 6–9. The removal of BPA by free HRP/GOx was substantially lower than that of the biocatalytic membrane under acidic (pH ≤ 5) or alkaline (pH ≥ 10) conditions. This suggests that the adsorption of HRP/GOx in COF provided effective protection for the enzyme. In the HRP/H_2_O_2_ system, BPA removal was lower, reaching only 74.51% even at the optimal pH. In the dual-enzyme system, the optimal pH for the two enzymes differed. The optimal pH for free GOx was ~5, where the maximum amount of H_2_O_2_ could be produced, while for free HRP, the optimal pH was ~7, where HRP would consume more H_2_O_2_. There was an extensive hydrogen bonding network at both the distal and proximal ends of HRP [45]. At pH 5, protonation of key residues in the sheme’s hydrogen bonding network took place, primarily due to the protonation of the distal histidine (pKa ≈ 5), which subsequently destabilized the heme structure [46]. Therefore, in acidic environments, although GOx remained highly active, the stability of HRP was reduced, limiting the overall efficiency of pollutant removal. In alkaline environments, the activity of both enzymes was severely inhibited, leading to a reduction in H_2_O_2_ content and electron-donating capacity. However, the gluconic acid produced in the dual-enzyme system enhanced resistance to the alkaline environment [47]. Owing to the continuous release of H_2_O_2_, the dual-enzyme system maintained a high BPA removal rate even in an alkaline environment. In summary, the self-regulating ability of the dual-enzyme system was unique compared with the single-enzyme system and effectively alleviated the inhibition caused by the alkaline environment.

**Figure 11:**
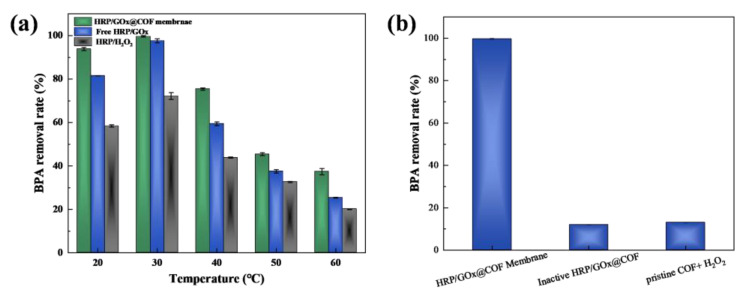
(**a**) BPA removal rate of HRP/GOx@COF membrane, free HRP/GOx and HRP/H_2_O_2_ at different temperature. (**b**) BPA removal rate of HRP/GOx@COF membrane, inactive HRP/GOx@COF and pristine COF+H_2_O_2_. (BPA: 10 mg/L, glucose: 5 mM, temperature: 25 °C, pH: neutral).

In wastewater treatment, the concentration of MPs fluctuated. As shown in Figure 10b, the biocatalytic membrane effectively removed BPA in the range of 10–100 mg/L, with a removal rate exceeding 90%. In the concentration range of 2–8 mg/L, the removal rate of BPA by the biocatalytic membrane gradually increased from 50% to 86.9%, indicating the limited removal rate under lower BPA concentrations. For the free HRP/GOx system, although the removal rate could reach up to 90% when the BAP concentration was 20 or 40 mg/L, the removal rate for both high and low concentrations of BPA was relatively low. In the single-enzyme system, the BPA removal rate was generally lower than in the dual-enzyme system. The single-enzyme system could not continuously release H_2_O_2_, making it susceptible to inhibition from excess H_2_O_2_ at high concentrations. In contrast, the HRP/GOx dual-enzyme system achieved a removal rate of more than 80% even under high BPA concentrations. Considering the temperature may also influence BPA removal performance of biocatalytic membrane by affecting enzyme activity, the effects of reaction temperatures were also investigated, and as shown in Figure 11, the effect of reaction temperatures was also investigated, and as shown in Figure 11a, the highest removal rate of all the systems was obtained at 30 °C. The observed decline in BPA removal efficiency at elevated temperatures can be attributed to enzyme denaturation and the consequential loss of catalytic activity. To gain deeper kinetic insight into the cascade system, the initial reaction rate for BPA degradation was calculated based on the concentration-time curve (Figure S4). The HRP/GOx@COF system exhibited a high initial rate (v_0_) of 2.5 μM min^−1^. Based on the amount of active HRP immobilized, an apparent turnover frequency (TOF) of ~0.8 s^−1^ was calculated, indicating efficient substrate conversion at each catalytic site. This value is comparable to HRP in other immobilized systems, confirming that the COF environment does not impose severe mass transfer limitations. Moreover, these two enzymes were also immobilized on COFs individually (HRP@COF+GOx@COF), and the BPA removal rate in this case (86.5%) is also lower than that obtained by the co-immobilized system (98.5%) (Figure S5). To clearly investigate the individual contribution of enzymatic degradation and the adsorption by COF carrier, some control experiments were conducted. Firstly, the amount of removed BPA by the pristine COF (without enzymes), was comparable to that adsorbed by the COF, indicating the absence of inherent Fenton-like or catalytic sites within the framework. Secondly, a control sample with heat-denatured enzymes (inactive HRP/GOx@COF) was tested, also showing a pollutant removal comparable to the adsorption of the pristine COF, thus confirming that the observed high degradation efficiency is exclusively mediated by the enzymatic cascade and not by physical adsorption. Quantitative analysis of these controls indicates that adsorption accounts for less than 15% of the total pollutant removal, with the dominant mechanism (>85%) being the HRP-H_2_O_2_ catalyzed oxidation (Figure 11b).

#### 3.3.4. Removal of Different MPs by Biocatalytic Membranes

**Figure 12:**
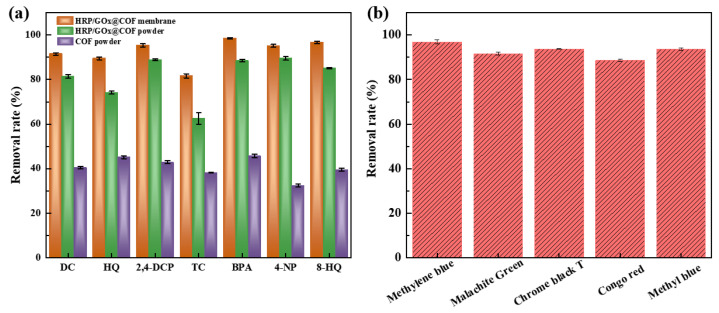
(**a**) Removal rate of different substrates by HRP/GOx@COF membrane, HRP/GOx@COF powder and COF powder (glucose: 5 mM, HRP: 4 mg, GOx: 4.5 mg, temperature: 25 °C, pH: neutral), (**b**) Removal rate of different dyes of HRP/GOx@COF membrane. (glucose: 5 mM, HRP: 4 mg, GOx: 4.5 mg, temperature:25 °C, pH: neutral).

To broaden the application range of dual-enzyme cascade catalytic membranes, the membranes were further tested for the removal of six other MPs: DC (50 mg/L), HQ (20 mg/L), 2,4-DCP (20 mg/L), TC (50 mg/L), 4-NP (20 mg/L), and 8-HQ (50 mg/L). As shown in Figure 12a, the biocatalytic membranes exhibited better removal rates for all seven MPs, with the removal rates of 2,4-DCP, 4-NP, and 8-HQ exceeding 95%. The removal of MPs by HRP/GOx@COF nanoparticles was mainly driven by adsorption and degradation, and its removal efficiency was lower than HRP/GOx@COF membrane. In addition, when using pristine COF, the removal rates of most MPs were only ~40%, relying mainly on the adsorption effect of the COF material itself. In summary, the combination of enzyme-catalyzed degradation, material adsorption, and membrane rejection contributed to the effective removal of MPs by biocatalytic membranes and enhanced their overall performance. While this study demonstrates the high catalytic capacity of the HRP/GOx@COF system, the pollutant concentrations tested (10–100 mg/L) are above typical environmental levels (ng/L-µg/L). This choice was primarily dictated by analytical constraints, as the detection limit of our HPLC-UV system (~0.1 mg/L) is insufficient for accurate kinetic measurements at trace concentrations. Therefore, this work serves as a vital proof-of-concept. More important steps, such as employing highly sensitive analytical techniques (e.g., LC-MS/MS) to monitor degradation kinetics at µg/L levels and evaluating the composite’s performance in complex matrices, will be the focus of subsequent research to translate this technology from the laboratory to real-world water treatment.

#### 3.3.5. Removal of Dyes by Biocatalytic Membranes

The dye removal rates by biocatalytic membranes were also evaluated, as shown in Figure 9b. The HRP/GOx@COF membrane removed more than 88% of the five dyes through a combination of degradation, adsorption, and rejection. The degradation mechanism of dyes by biocatalytic membranes was consistent with that of MPs. First, HRP was activated in the presence of H_2_O_2_ to generate HRP-I and water molecules. The active center of HRP-I contained oxyferryl groups surrounded by porphyrins in the form of π radical cations [48]. The generated HRP-I then oxidized dye molecules through redox reactions and produced dye radicals and HRP-II. Finally, HRP-II interacted with unreacted dye molecules to generate more dye radicals and regenerate HRP. Finally, the generated dye radicals combined through free radical coupling reactions [49] to form polymeric macromolecules, which could then be separated from water by the membrane. Moreover, the enhanced hydrophilicity of HRP/GOx@COF facilitates the adsorption of dissolved oxygen, which is a critical substrate for the GOx-catalyzed H_2_O_2_ generation process, thereby improving catalytic efficiency.

#### 3.3.6. Comparison of Hydroxyl Radicals in Dual-Enzyme and Single-Enzyme Systems

**Figure 13:**
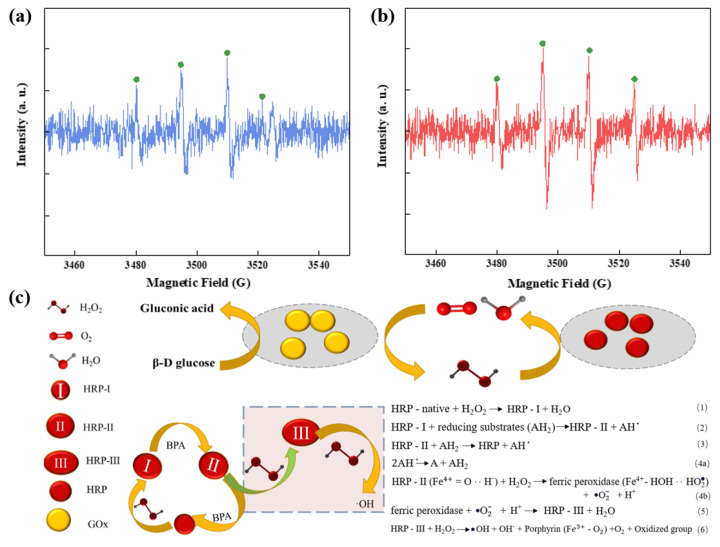
EPR spectra of DMPO^−^·OH in the HRP/GOx (**a**) and HRP/H_2_O_2_ (**b**) (BPA: 10 mg/L, glucose: 5 mM, HRP: 4 mg, GOx: 4.5 mg, H_2_O_2_: 3 mM, temperature: 25 °C, pH: neutral); (**c**) Mechanism of dual enzyme cascade catalysis.

Electron paramagnetic resonance (EPR) tests were conducted using DMPO as a spin-trapping agent [50]. As shown in Figure 13a,b, the DMPO^−^•OH signal (with a peak height ratio of 1:2:2:1) in the HRP/GOx system was significantly weaker than that in the HRP/H_2_O_2_ system [25]. According to literature, H_2_O_2_ can oxidize HRP-II to produce •O_2_^−^ and iron-containing peroxidase [51]. However, under acidic conditions, the iron-containing peroxidase and •O_2_^−^ will generate HRP-III, which lacks catalytic activity. Although HRP-III is not catalytically active, it can undergo a biological Fenton reaction with H_2_O_2_ to generate hydroxyl radicals [52]. Although hydroxyl radicals also play a role in pollutant degradation, they can cause significant damage to the enzyme’s active sites. Over time, this damage reduces the enzyme’s reusability and is detrimental to pollutant removal [53]. The higher concentration of hydroxyl radicals in the single-enzyme system indicated a more intense biological Fenton reaction. Therefore, the observed difference in the intensity of the DMPO^−^•OH adduct signal between the HRP/H_2_O_2_ and HRP/GOx systems provides key mechanistic insight Figure 13c into the efficiency of the cascade. The stronger signal in the HRP/H_2_O_2_ system is attributed to the high bolus addition of H_2_O_2_, which promotes side reactions leading to •OH generation. In contrast, the significantly weaker signal in the HRP/GOx cascade system indicates a highly efficient coupling between the enzymes. Here, GOx generates H_2_O_2_ in situ at a low, steady-state concentration, which is immediately and preferentially utilized by HRP in its primary catalytic cycle to oxidize the pollutant. This efficient substrate channelling minimizes the accumulation of H_2_O_2_ and thus reduces side reactions that generate free •OH radicals detectable by EPR. Consequently, the weaker EPR signal is not an indicator of lower activity but rather of a more productive and selective use of the generated H_2_O_2_, highlighting a key advantage of the co-immobilized cascade system.

#### 3.3.7. Reusability and Storage Stability

**Figure 14:**
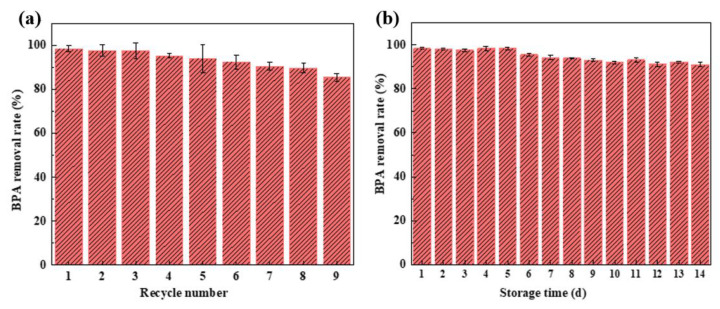
(**a**) Reusability and (**b**) storage stability of HRP/GOx@COF membrane (BPA: 10 mg/L, glucose: 5 mM, temperature: 25 °C, pH: neutral, storage temperature: −8 °C).

Figure 14a shows that the BPA removal rate of the HRP/GOx@COF membrane remained at 85% after nine consecutive cycles. The excellent reuse stability of the HRP/GOx@COF membrane further demonstrated that the pre-adsorption immobilization strategy effectively prevented enzyme leakage and inactivation during use. The decrease in removal rate may be due to the accumulation of polymer produced by the oxidation of MPs on the surface of the material. Figure 14b shows the storage stability of the HRP/GOx@COF membrane. The BPA removal rate by the biocatalytic membrane remained around 98% for the first five days. After that period, the removal rate began to decrease slightly but remained above 90% after 14 days. The results showed that the HRP/GOx@COF biocatalytic membrane exhibited excellent storage stability. The enzymes confined within the ordered and stable COF structure retained strong stability and biological activity. While the batch-wise reusability tests and storage stability demonstrate promising robustness, assessing performance in a continuous-flow system would be essential for practical deployment; however, the current HRP/GOx@COF system and operation are not directly suitable for continuous-flow membrane reactors. Therefore, future work will focus on evaluating its long-term stability and efficiency in a continuous-flow mode using simulated and real wastewater matrices.

## 4. Conclusions

In this study, HRP and GOx were successfully immobilized in a COF through self-healing and self-crystallization, enabled by the dynamic covalent chemical properties of COF. This strategy not only maintains the activity but also enhances the stability of enzymes. The HRP/GOx@COF biocatalytic membrane was successfully prepared through the loading of the immobilized enzymes onto a substrate membrane, using dopamine for adhesion. The effects of dual enzyme amount and glucose concentration on the BPA removal rate of the biocatalytic membrane were investigated. At an HRP dosage of 4 mg, a GOx dosage of 4.5 mg, and a glucose concentration of 5 mM, the BPA removal rate reached 99%. Additionally, the removal efficiency of DC, 2,4-DCP, and 8-HQ exceeded 95%. The removal rate of five dyes with different properties also exceeded 88%. Furthermore, the BPA removal rate of the HRP/GOx@COF biocatalytic membrane remained high under both acidic and basic conditions. Confining the enzyme within an orderly and stable COF resulted in excellent stability for the biocatalytic membrane, which maintained a BPA removal rate of 85% after eight cycles and over 90% after 14 days of storage.

## Data Availability

Data underlying the results presented in this article are not publicly available at this time but may be obtained from the authors upon reasonable request.

## Supplementary Materials

The following supporting information can be downloaded at https://www.mdpi.com/article/10.3390/nano15181431/s1: Figure S1: CLSM images of aCOF and COF in optical; Figure S2: 3D and 2D AFM images of (a) HPAN substrate, (b) PDA@HPAN membrane and (c) HRP/GOx@COF membrane; Figure S3: Water permeance of COF, GOx@COF, HRP@COF and HRP/GOx@COF; Figure S4: The concentration-time curve for the BPA degradation over HRP/GOx@COF membrane under the optimal conditions (pH = 7 and BPA concentrations is 10 mM); Figure S5: BPA removal rate of free HRP/GOx, HRP/H_2_O_2_, HRP@COF+ GOx@COF and HRP/GOx@COF membrane under the optimal conditions (pH = 7 and BPA concentrations is 10 mM). Table S1: BET surface area, pore volume and pore diameter of different samples; Table S2: Surface roughness parameters of membranes.
