# Supplementary material for: Covalent Organic Framework-Based Nanomembrane with Co-Immobilized Dual Enzymes for Micropollutant Removal
Source: Nanomaterials (Basel). 2025 Sep 18;15(18):1431. doi: 10.3390/nano15181431 (PMC12472217; doi:10.3390/nano15181431)
Supplement: Supplementary file 1 [file nanomaterials-15-01431-s001.zip › nanomaterials-3825602-supplementary.pdf]

nanomaterials

Supporting Information

Covalent Organic Framework-Based Nanomembrane with Co-Immobilized Dual Enzymes for Micropollutant Removal

Junda Zhao [1], Guanhua Liu [2], Xiaobing Zheng [2], Liya Zhou [2], Li Ma [2], Ying He [2,*] and Xiaoyang Yue [2,*] and Yanjun Jiang [2]

1 Arizona College of Technology, Hebei University of Technology, Tianjin 300401, China; jundazhao@arizona.edu
2 School of Chemical Engineering and Technology, Hebei University of Technology, Tianjin 300401, China;
* Correspondence: heying1980@hebut.edu.cn; xiaoyang.yue@hebut.edu.cn

Methods

1. Characterization

A confocal laser scanning microscope (Leica, TCS SP5) was used to observe the distribution of enzymes. The surface and cross-sectional morphology of the membranes were observed using scanning electron microscopy (SEM, FEI, Nano SEM450). The internal structure of the prepared materials was examined via transmission electron microscopy (TEM, FEI, Talos F200S). Qualitative protein characterization was performed via Sodium dodecyl sulfate polyacrylamide gel electrophoresis (SDS-PAGE) gel electrophoresis. The hydrophilicity of the membranes was evaluated through the measurement of water contact angles (WCAs) using an optical contact angle tester (German KRUSS, DAS30).

2. SDS-PAGE Analysis

Assemble the electrophoresis apparatus and check for leaks. In a fume hood, prepare the separation gel with acrylamide, Tris-HCl (pH 8.8), SDS, APS, and TEMED, pour to the desired height, and cover with anhydrous ethanol to level and promote polymerization. After solidification, remove the ethanol. Prepare the concentration gel with acrylamide, Tris-HCl (pH 6.8), SDS, APS, and TEMED, pour above the separation

gel, insert the comb, and allow to solidify. Dilute the 10X electrophoresis buffer to 1X, fill the tank, and ensure the wells are covered. Mix the sample with loading buffer containing mercaptoethanol, heat at 95°C for 5 minutes, centrifuge if needed, and load the sample. Run electrophoresis at 105V for 20 minutes, then 155V for 90-120 minutes. Afterward, stain with Coomassie Brilliant Blue for 40-60 minutes and decolorize until the background clears.

3. Enzyme activity assay

GOx activity assay

The activity of GOx was measured by monitoring the change in the absorbance of H_2O_2 at 351 nm using SpectraMax190 (Molecular devices, America). The activity assay was carried out in 1 mL reaction mixture containing citric acid-sodium citrate buffer (100 mM, pH 6.0), 10 mM glucose and 0.1 mg/mL free GOx (or immobilized GOx: 4mg/ml) at 25 °C for 1.0 min. Then 0.3 mL of iodide reagent (0.4 M potassium iodide, 0.05 M NaOH, 10 mM ammonium molybdate) and 0.3 mL of 0.5 M potassium hydrogen phthalate was added to 0.4 mL reaction sample solution (filtered through a PVDF syringe filter (0.2 μm, Roth) to remove the immobilized enzyme particles). After intensely shake at 25 °C for 3 seconds, measured at 351 nm using SpectraMax190 (Molecular devices, America). One unit activity was defined as the amount of enzyme that catalyzed the generation of 1 μmol H_2O_2 during 1 min. All measurements were carried out in triplicate.

HRP activity assay

The definition of horseradish peroxidase enzyme activity is :1U refers to the amount of enzyme required to oxidize 1μmol of guaiacol within 1 minute at 37°C. First, mix 2 mL of H_2O_2 (3.0 mM), 2 mL of guaiacol (15 mM), and 1mL of PBS buffer solution (pH =6) and shake. Take 1 mL of the mixed solution and measure its absorbance value A_0 at 470 nm. A certain amount of HRP was added to the above-mentioned mixed solution. After

reacting for 2 minutes, 1mL of the solution was taken to measure its absorbance value A_1 at 470 nm. The formula for calculating enzyme activity U is as follows:

$$U = \frac{A_1 - A_0}{T \times \tau \times M} \times V$$

Here, T represents the reaction time (min), is the molar absorption coefficient (26.6 mL μmol^{-1} cm^{-1}), and M is the actual dosage of the enzyme (mg).

Table S1 BET surface area, pore volume and pore diameter of different samples

Sample	BET Surface area(m^2/g)	Pore diameter (nm)
aCOF	80.7828	10.44
HRP/GOx@aCOF	15.9895	8.1
COF	337.7087	5.5
HRP/GOx@COF	213.2290	4.4

Table S2 Surface roughness parameters of membranes

Sample membrane	Rq (nm)	Ra (nm)
HPAN	43	34.9
PDA@HPAN	50	46.6
HRP/GOx@COF	103	85.1

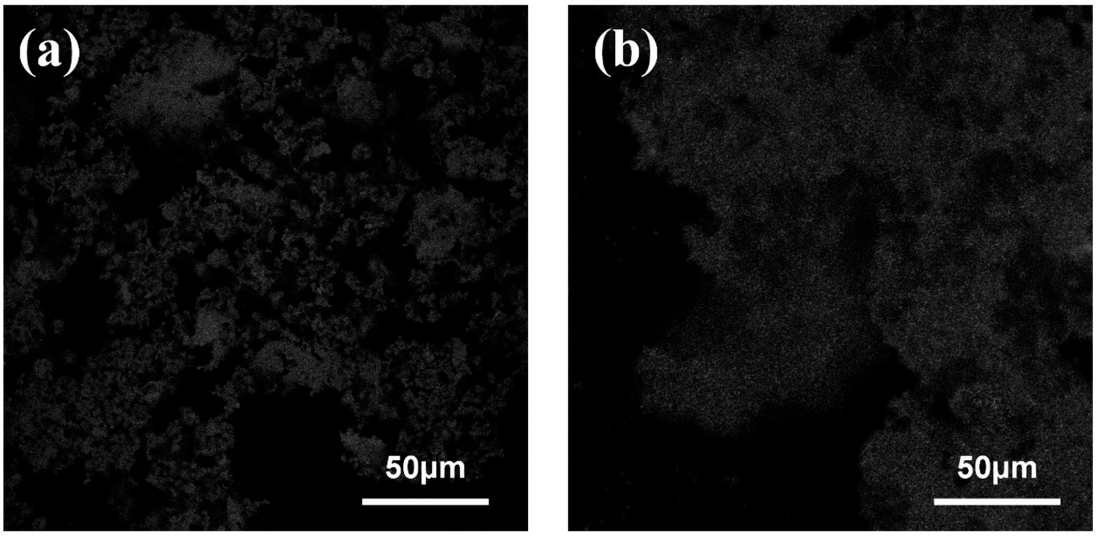

Figure S1. CLSM images of aCOF and COF in optical

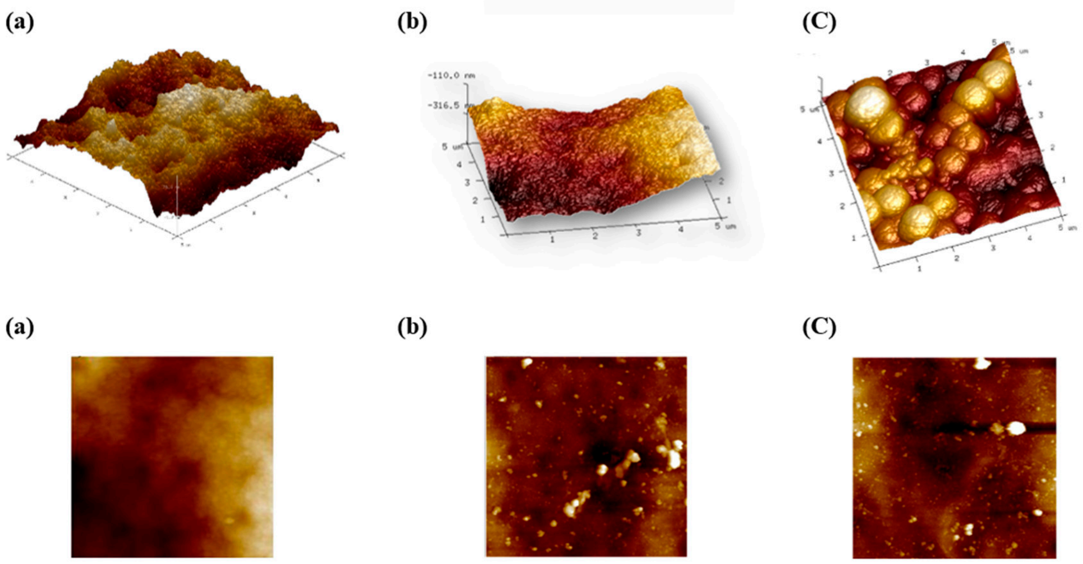

Figure S2. 3D and 2D AFM images of (a) HPAN substrate, (b) PDA@HPAN membrane and (c) HRP/GOx@COF membrane

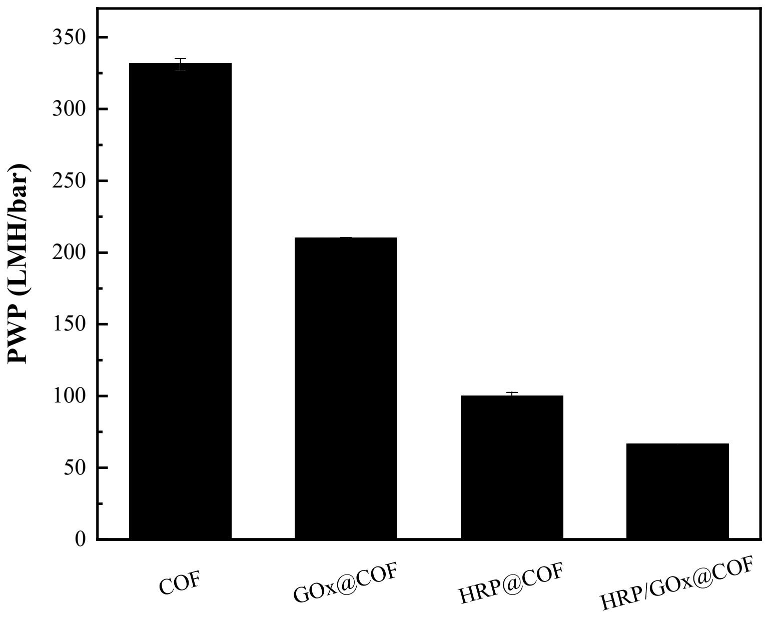

Figure S3. Water permeance of COF, GOx@COF, HRP@COF and HRP/GOx@COF

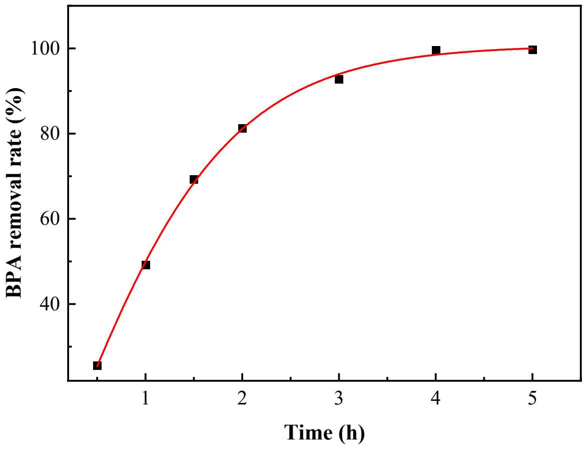

Figure S4. The concentration-time curve for the BPA degradation over HRP/GOx@COF membrane under the optimal conditions (pH=7 and BPA concentrations is 10 mM).

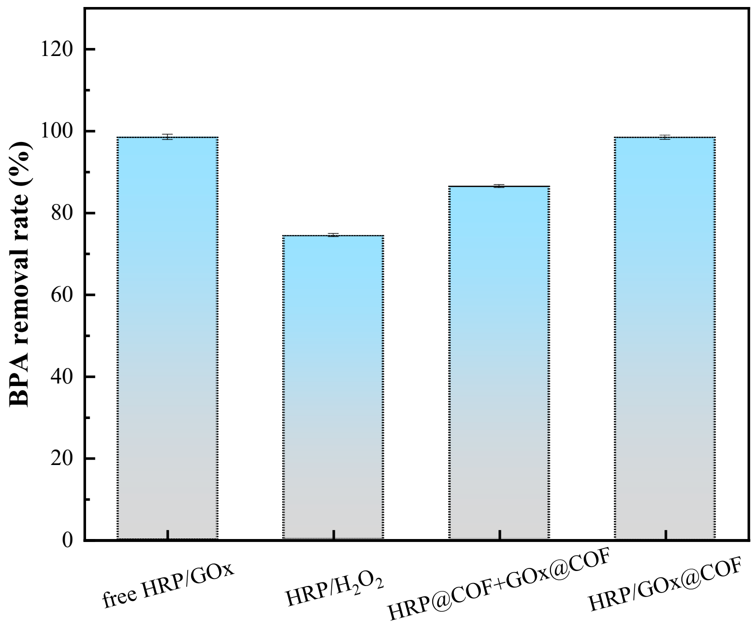

Figure S5. BPA removal rate of free HRP/GOx, HRP/H_2O_2, HRP@COF+ GOx@COF and HRP/GOx@COF membrane under the optimal conditions (pH=7 and BPA concentrations is 10 mM).
